# Investigating the potential role of non-*vls* genes on linear plasmid 28–1 in virulence and persistence by *Borrelia burgdorferi*

**DOI:** 10.1186/s12866-016-0806-4

**Published:** 2016-08-08

**Authors:** Petronella R. Hove Magunda, Troy Bankhead

**Affiliations:** 1 Department of Veterinary Microbiology and Pathology, Washington State University, Pullman, WA USA; 2 Paul G. Allen School for Global Animal Health, Washington State University, Pullman, WA USA

**Keywords:** *Borrelia*, Lyme disease, Linear plasmid 28–1, Persistence, Immune evasion

## Abstract

**Background:**

The lp28-1 plasmid is required for persistent infection by the Lyme disease spirochete, *Borrelia burgdorferi*. Mutational studies on this plasmid have shown that the *vls* locus is important for antigenic variation of the VlsE lipoprotein that leads to immune evasion and persistence. However, it is still unknown whether the *vls* system is the only genetic locus on this plasmid necessary for long-term infection, and thus the potential role of non-*vls* genes on lp28-1 in virulence and persistence is yet to be fully determined. Despite extensive mutational analyses, two lp28-1 regions containing the ORFs *bbf19* - *bbf22* and *bbf27* – *bbf30* have not yet been mutated in their entirety.

**Results:**

In this study, we set out to establish if these unstudied regions of lp28-1 play a role in spirochete persistence. Results show that the generated mutants were fully infectious in immunocompetent mice, and were able to persist for 91 days following infection. Following this finding, *ospC* expression by these mutants was determined, as it has been reported that spirochetes lacking lp28-1 fail to downregulate expression of this lipoprotein leading to immune clearance. Data presented here failed to show a definitive difference in *ospC* expression levels during host infection when the mutants were compared to the wild type.

**Conclusions:**

Overall, the results strongly suggest that non-*vls* genes residing on lp28-1 do not play a role in spirochete persistence during infection of the mammalian host, and that the regions under study are likely not involved in the regulation of *ospC* expression. In conjunction with previous studies involving mutation of non-*vls* loci on lp28-1, these findings suggest that the *vls* locus is likely the sole genetic element on this plasmid responsible for immune evasion and persistence exhibited by the Lyme disease pathogen.

## Background

**Fig. 1:**
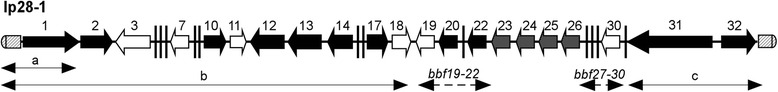
Schematic of the open reading frames of the *B. burgdorferi* lp28-1 plasmid. Genes are flanked by telomeres (striped regions). Double-headed solid arrows show regions that have been previously deleted: **a** [11], **b** and **c** [8]. Genetic loci *bbf19-22* or *bbf27-30* were chosen for mutational analysis, as there is no knowledge for their respective roles in *B. burgdorferi* persistence. Conserved regions (*black*); pseudogenes (*white*); short genes less than 300 bp (vertical lines); autonomous replication genes (grey)

Lyme disease is caused by infection with the tick-transmitted spirochete, *Borrelia burgdorferi*. It is currently the most common vector-borne disease in the northern hemisphere, occurring in parts of North America, Europe and Asia [1]. Persistent host infection by *B. burgdorferi* has been linked to the presence of the 28 kilobase linear plasmid, lp28-1, which harbors 32 open reading frames that are designated *bbf01* to *bbf32* ([2, 3]; Fig. 1). Clones lacking lp28-1 exhibit an intermediate infectivity phenotype, whereby these spirochetes are able to disseminate to tissue sites but are unable to persist in the murine host [4–6]. Notably, these same clones are capable of long-term survival in severe-combined immunodeficient (SCID) mice that lack an effective antibody response [4, 7]. Studies involving telomere-mediated deletion of a 10 kb region (*bbf31*-*bbf32*) containing the VMP-like sequence (*vls*) locus responsible for antigenic variation of a *vls*-expressed (VlsE) lipoprotein found that these mutant spirochetes are cleared in immunocompetent mice by day 21 post infection, matching the phenotype observed with the complete loss of lp28-1 [8, 9].

Although numerous studies have been conducted on the importance of the *vls* locus, it has not been fully determined whether other genes on lp28-1 play a role in infectivity and persistence. Mutation or deletion of the *bbf01* gene that encodes for the arthritis related protein, Arp, was shown to exhibit a reduced ability to cause joint inflammation, but did not alter infectivity or persistence in immunocompetent mice [10, 11]. Clones containing a deletion of *bbf01* to *bbf18* were found to be fully infectious and persistent following infection in immunocompetent mice [8]. However, signature-tagged transposon mutagenesis of lp28-1 [12] identified several genes within the *bbf1-18* region (*bbf03, bbf05, bbf10,* and *bbf18*) as having a possible impact on infectivity. Region *bbf23-bbf26* of lp28-1 codes for the paralogous family proteins 49, 32, 50 and 57, respectively. These genes are known to be required for autonomous replication of the lp28-1 plasmid [13], and have been shown to be required for infectivity [12]. In addition to previous mutational studies, work by Embers et al*.* found that regulation of outer surface protein C (OspC) is impaired in spirochetes lacking lp28-1, which could also potentially contribute to the lack of persistence exhibited by this strain [14]. This disregulation resulted in continued expression of OspC, which has been shown to lead to spirochete clearance by an adaptive host immune response [15–18].

To date, with the exception of *bbf29* [19], two regions containing the ORFs *bbf19* - *bbf22* and *bbf27* – *bbf30* have not been mutated in their entirety. Hence, the question addressed in this study is whether loss of the genes within these two regions has an effect on infectivity and persistence by *B. burgdorferi.* The *bbf19-22* region encodes one pseudogene (*bbf19*), a conserved hypothetical protein (*bbf20*), a short gene (*bbf21*) and a putative p23 protein (*bbf23)*. Region *bbf27-30* encodes mostly short genes (*bbf27-29*) and one pseudogene (*bbf30*) [20]. To further investigate the role of non-*vls* genes of lp28-1 in disease persistence and potentially the regulation of *ospC* expression, mutants were generated that lacked either region *bbf1-18*, *bbf19-22* or *bbf27-30,* and their respective impact on infectivity and disease persistence was assessed in immunocompetent C3H mice. Results provided here show no significant difference in infectivity and persistence between wild type *B. burgdorferi* and the generated mutants. In addition, the particular lp28-1 regions under study may not be involved in OspC downregulation as a mechanism of immune avoidance, as there was no significant difference in *ospC* expression levels during host infection between the wild-type and mutant clones.

## Methods

### *B. burgdorferi* clones and culture methods

*Borrelia burgdorferi* B31-A3 (wild type) was a kind gift from Patti Rosa [21]. Clones described in this study were generated from the above-mentioned B31 clone (Table 1). All *B. burgdorferi* clones were cultivated in liquid BSK-II medium supplemented with 6 % rabbit serum (Cedarlane Laboratories, Burlington, NC) and incubated at 35 °C under 2.5 % CO_2_. Mutant strains were grown in media supplemented with kanamycin (200 μg/ml). Cell densities and growth phase were monitored by dark-field microscopy and enumerated using a Petroff-Hausser counting chamber. Blood and tissue samples were cultured in BSK-II supplemented with 6 % rabbit serum and containing *Borrelia* antibiotic cocktail (0.02 mg/ml phosphomycin, 0.05 mg/ml rifampicin and 2.5 μg/ml amphotericin B).

**Table 1:** *B. burgdorferi* clones used in this study

*B. burgdorferi* B31 clone	Missing plasmids	Reference
A3 wild type	cp9	[21]
A3∆*bbf 1–18* (*Bb*Δ*1-18*)	cp9	[8]
A3∆*bbf 19–22* (*Bb*Δ*19-22*)	cp9	This study
A3∆*bbf 27–30* (*Bb*Δ*19-22*)	cp9	This study
5A8 (*Bb*Δ*lp28-1*)	lp28-1	[6]

### Generation of lp28-1 mutant clones

The mutant lacking regions *bbf1-18* was regenerated through targeted deletion as described in a previous publication [8]. For deletion of *bbf19-22* and *bbf27-30* from lp28-1 by allelic exchange, a 500 bp sequence upstream and downstream of each region to be deleted was PCR amplified from the annotated lp28-1 sequence (NCBI Reference Sequence: NC_001851.2 http://www.ncbi.nlm.nih.gov/) using primers listed in Table 2. The resulting DNA product was then cloned into the pJET 2.1 plasmid vector (Fermentas, USA). Primers annealing within the 500 bp region were then used to amplify the DNA vector excluding the regions to be deleted. An insert containing the *aphI* gene conferring resistance to kanamycin driven by the *flgB* promoter of *B. burgdorferi* was then ligated to this PCR product to create the plasmid DNA construct for allelic exchange (pPH17 or pPH30 for the deletion of *bbf27-30* and *bbf19-22*, respectively). The resulting plasmid was then transformed into *Escherichia coli* DH5α competent cells. Plasmid DNA isolated from individual *E. coli* clones was verified for correct size and orientation by restriction digest before transformation into *B. burgdorferi* cells.

**Table 2:** Primers used in this study

P330	GTCTGTGGTAGTTACTAGTTACTTTAAATACC	Forward primer for *bbf27-30* target
P331	CCGAAATATTCCTATCTACTTAACAAC	Reverse primer for *bbf27-30* target
P332	CCGGCCGGCGAATTTTGAGTCCTCTAGTGAGTTGTG	Left primer for inverse PCR of *bbf27-30* target in pJET with NgoMIV site
P333	CCGGCTAGCGTTATAAGCCCTCCATTTGATAATTTTTTG	Right primer for inverse PCR of *bbf27-30* target in pJET with NheI site
P366	CTTAATTTGTGACCGCCATTAGAGC	Forward primer for *bbf27-30* screen
P367	GGGTTTTTTGAAACAAATCTTGC	Reverse primer for *bbf27-30* screen
P411	GAGTTTCTGGTAAGATTAATGCTC	Forward primer for *flaB* RT-qPCR
P412	CATTTAAATTCCCTTCTGTTGTCTGA	Reverse primer for *flaB* RT-qPCR
P413	AGAGGTTTGTCACAAGCTTCTAGAAATACTTCAAAGGC	*flaB* RT-qPCR probe
P482	GAACAAGCTGAAAAATATAAAAAAGTAATG	Forward primer for PCR amplification of *bbf19-22* target
P483	CTGGTTACTTTTTAGATAGAGTTTTTATAGAG	Reverse primer for PCR amplification of *bbf19-22* target
P484	CCGGCCGGCGGTTTAGACTTGCATTTA TATCTCC	Left primer for inverse PCR *bbf19-22* target in pJET with NgoMIV site
P485	CCGGCTAGCCCCCTCCTTATATTTTTTTATATATAAAAG	Right primer for inverse PCR of *bbf19-22* target in pJET with NheI site
P486	GCTTATAAGCTTTATTAACACCCATATATTC	Forward primer for *bbf19-22* screen
P487	CCCGCGAGGTATATTTATTTATATTG	Reverse primer for *bbf19-22* screen
P709	TTACGGATTCTAATGCGGTTT	Forward primer for *ospC* RT-qPCR
P710	TTTACCAATAGCTTTAGCAGCAA	Reverse primer for *ospC* RT-qPCR
P711	TGTGAAAGAGGTTGAAGCGTTGCTG.	*ospC* RT-qPCR probe
P828	CTGCACTACCACAAGAGATTGCA	Forward primer for PCR screen for left-end sequence of lp28-1
P829	CTCTTCTCCTCTCTTCTTCTCTCT	Reverse primer for PCR screen for left-end sequence of lp28-1
P830	CATTTCTAGTCTAGATTGCAGTTATTTCTAAAATTAACT	Forward primer for PCR amplification for lp28-1 left-end deletion target
P831	GTGCCCAGGCGGCCGTCCTTATTCTTCTGGCATAGAAGT	Reverse primer for PCR amplification for lp28-1 left-end deletion target

### *B. burgdorferi* transformation

*B. burgdorferi* B31-A3 wild-type cells were electroporated and cultured as previously described [8, 22]. DNA from culture-positive wells was extracted using a DNeasy Blood and Tissue Kit (Qiagen, Germantown, MD), and used for PCR analysis to confirm the presence of the antibiotic-resistance gene and the absence of *bbf1-18, bbf19-22* or *bbf27-30* utilizing primer sets described in Table 2. Plasmid content for each verified transformant was determined by PCR using plasmid-specific primers as previously described [6].

### Southern Blot analysis

Total plasmid DNA was extracted from *B. burgdorferi* clones using the Plasmid Midi Kit (Qiagen) and separated on a 1 % agarose gel at 80 V for 23 h (250 ng of DNA was used per lane). DNA was then transferred onto a nylon membrane and hybridized with Digoxigenin (DIG)-labeled probes following manufacturer’s guidelines (Roche, Indianapolis, IN).

### Infection of mice

Three to four week-old male C3H/HeN mice (Harlan, Indianapolis, IN) were infected by subcutaneous needle inoculation with 10^5^ total spirochetes per dose. *B. burgdorferi* clones from frozen glycerol stock were passaged no more than two times *in vitro* prior to use in mouse infection assays. Infection was monitored by culturing either blood samples or ear biopsies at the indicated times post infection. After infection, mice were monitored for presence of infection at day seven by blood collection. Tissues for RT-qPCR analysis were collected at days 14, 21, 56 and 91. Two ear punch biopsies of about 2 mm diameter were collected from each mouse and stored in RNA later in −20 °C until RNA extraction was done.

### RNA extraction and gene expression analysis by droplet digital PCR

RNA was extracted from the tissues using the RNeasy® Fibrous Tissue Mini Kit (Qiagen, Valencia, CA) and treated with the RNase-free DNase set (Qiagen) to remove any residual DNA. The concentration, purity and integrity of RNA were determined electrophoretically and by measuring the absorbance. The reverse transcription reaction was performed in a total volume of 20 μL using iScript Advanced cDNA Synthesis Kit for RT-qPCR (Biorad) following manufacturers guidelines. A quantity of 100 ng total RNA was used as template. Following generation of cDNA, droplet digital PCR was performed using the QX100 Droplet Digital PCR (ddPCR™) system (Bio-Rad). The *ospC* and *flaB* genes were amplified using primers and probes shown in Table 2. The primer probe mixture, cDNA and 1x ddPCR supermix were pipetted into the DG8™ Cartridge for droplet formation in the QX100 droplet generator. Droplets were then transferred into a 96 well PCR plate and heat-sealed with a foil plate seal (Biorad) before placement into the C1000 Touch Thermal Cycler. Reactions were performed in biological replicates in a total volume of 40 μl. Cycling parameters (with ramping speed at 2 °C s^−1^) were as follows: enzyme activation was done at 95 °C for 10 min followed by a denaturation for 30 s at 94 °C; annealing and extension for 1 min at 60 °C was repeated for 40 cycles. After PCR, the plate was placed in the QX100 droplet reader and relative gene expression determined using the QuantaSoft software.

### Statistical analysis

SigmaPlot 11.0 software was used for all statistical data analysis. Normalized *ospC* transcript values from mice infected with the mutant clones were compared to transcript levels from mice infected with the wild type clone on respective days tested. Results were analyzed by the Student’s *t*-test and *p* values ≤0.05 were considered statistically significant.

## Results

### Generation of lp28-1 mutant *B. burgdorferi* clones

**Fig. 2:**
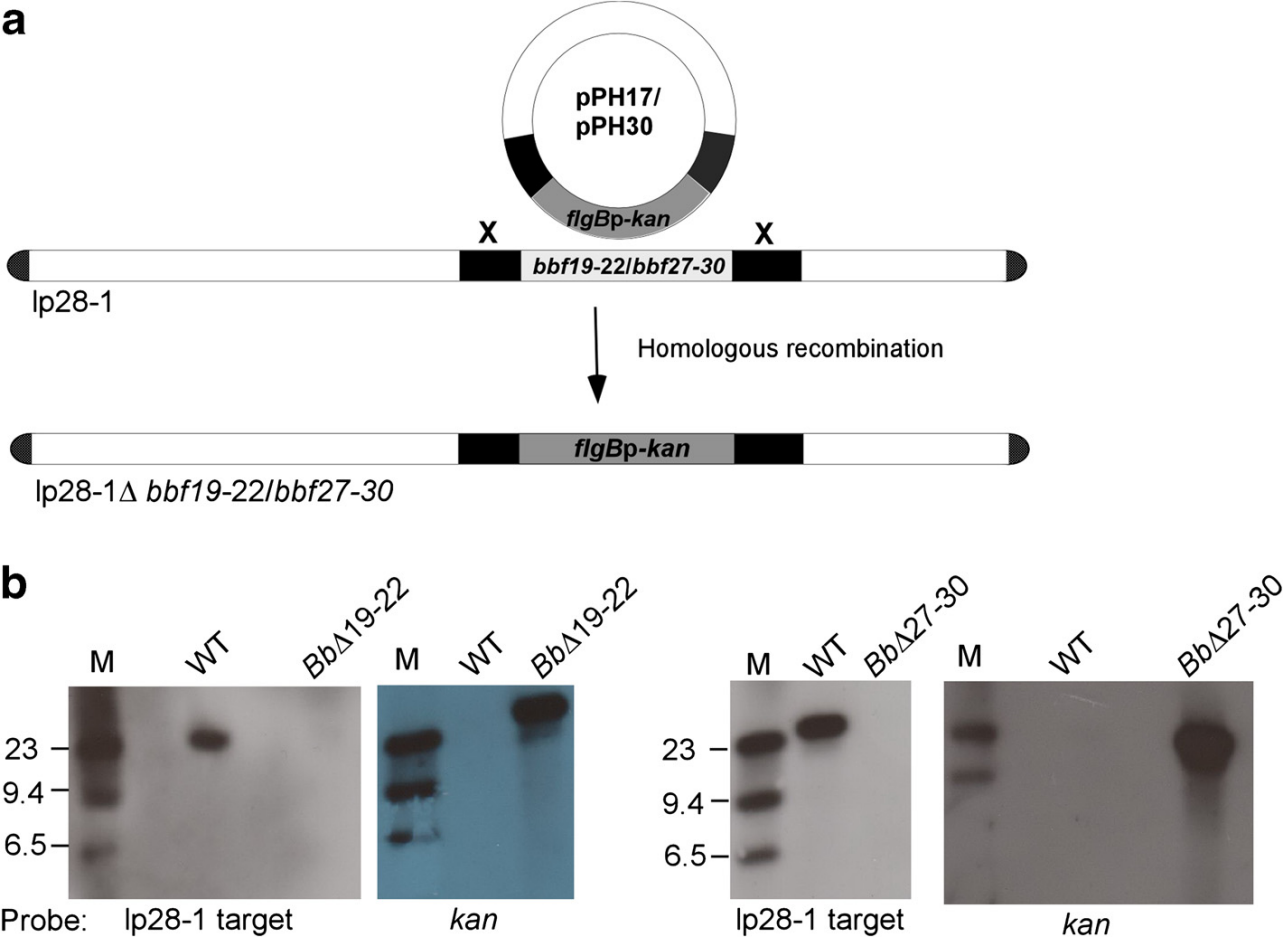
Generation and verification of lp28-1 mutant clones of *B. burgdorferi*. **a** Schematic of the construction strategy for the lp28-1 mutant clones *Bb∆19-22* and *Bb∆27-30* by allelic exchange is shown. *Bb∆1-18* was generated by targeted telomere deletion as previously described in a published study [8]. To confirm the loss of regions *bbf19-22* or *bbf27-30*, DNA was isolated and subjected to Southern blot analysis (**b**). All mutant clones provided a positive signal of the expected size (~28 kb) when probed for the *kan* cassette. The blot also confirmed the absence of the targeted region in the two mutant clones. WT, wild type; M, DNA size marker

Genetic loci of the lp28-1 plasmid containing the genes *bbf19-22* and *bbf27-30* (see Fig. 1) were chosen for mutational analysis to assess their respective roles in *B. burgdorferi* persistence. Deletion of each genetic region was achieved using allelic exchange as shown in Fig. 2a. Deletion constructs (pPH17 and pPH30) were generated that carried a kanamycin-resistance gene (*kan*) driven by a *B. burgdorferi flgB* promoter flanked by 500 bp homologous sequences upstream and downstream of the targeted region. Genetic deletion of the *bbf19-22* and *bbf27-30* regions was achieved by insertion of the selectable *kan* marker by homologous recombination to generate the mutant clones, *Bb∆19-22* and *Bb∆27-30*, respectively. Transformants were PCR screened for the presence of the kanamycin-resistance cassette, and clones meeting this criterion were then further analyzed for their total plasmid profile. All clones contained the full plasmid profile, with the exception of cp9. The cp9 plasmid is normally absent from the parental B31-A3 clone, and is not necessary for infection or pathogenesis [21]. To confirm the loss of each region, DNA was isolated from *Bb∆19-22* and *Bb∆27-30* and subjected to Southern blot analysis (Fig. 2b). A *B. burgdorferi* mutant lacking *bbf1-18* was also generated and verified as previously reported [8]. This mutant was included because previous infection studies were only carried out for 28 days, and the intention of this study was to prolong infection for 91 days to assess for long-term infection.

### Lp28-1 mutant clones are capable of dissemination and long-term infection in immunocompetent mice

To assess the individual infectivity profiles of the newly generated mutants, groups of six C3H/HeN (C3H) mice each were needle inoculated with either *Bb∆1-18, Bb∆19-22* or *Bb∆27-30* clones. Two groups of three C3H mice each were infected with wild type or lp28-1-deficient (*Bb*Δ*lp28-1*) *B. burgdorferi* clones to serve as positive and negative controls, respectively. As shown in Table 3, blood samples collected at day 7 post infection produced positive cultures for spirochetes in all inoculated mouse groups except with the *Bb∆19-22* mutant. Positive blood cultures were obtained from only 3 out of 6 mice infected with the *Bb∆19-22* clone. This pattern was also observed for this same group of mice at day 14 after culture of ear biopsies, suggesting a possible delay in dissemination to this tissue site. At day 14 post infection, cultures were positive for spirochetes in mice infected with wild type, *Bb∆1-18* and *Bb∆27-30*. As expected, spirochetes could not be detected from tissues collected from day 14 onwards from mice infected with the *Bb*Δ*lp28-1* clone due to the lack of lp28-1 [8]. Long-term infection (>21 days) was conducted to assess whether the *Bb∆1-18, Bb∆19-22* and *Bb∆27-30* mutant clones would eventually be cleared in infected mice. Ear biopsies were collected at days 21 and 56 post infection, and mice were sacrificed at day 91 to harvest heart, bladder, ear, and joint tissues for spirochete culture. Ear biopsies and cultured tissues from all mice were found to be culture positive, demonstrating that the *Bb∆1-18, Bb∆19-22* and *Bb∆27-30* clones were fully capable of dissemination and long-term persistent infection (Table 3).

**Table 3:** Infectivity of *B. burgdorferi* clones in C3H mice

Days post infection:								
	7	14	21	56	91			
*B. burgdorferi* clone	Blood	Ear			Heart	Bladder	Ear	Joint
Wild type	3/3^*^	3/3	3/3	3/3	3/3	3/3	3/3	3/3
*Bb∆1-18*	6/6	6/6	6/6	6/6	6/6	6/6	6/6	6/6
*Bb∆19-22*	3/6	3/6	6/6	6/6	6/6	6/6	6/6	6/6
*Bb∆27-30*	6/6	5/6	6/6	6/6	6/6	6/6	6/6	6/6
*Bb*Δ*lp28-1*	3/3	1/3	0/3	0/3	0/3	0/3	0/3	0/3

^*^Values listed correspond to numbers of positive cultures/number of mice tested. Six or three mice were used for each *B. burgdorferi* clone

### Lp28-1 mutants are capable of downregulating *ospC* expression during host infection

Associated with the development of the host-acquired immune response is OspC downregulation and increased synthesis of VlsE on lp28-1 [23, 24]. To determine if the deletion of *bbf1-18, bbf19-22* or *bbf27-30* has an effect on *ospC* expression during infection of a murine host*,* groups of 3 C3H mice each were needle inoculated with 10^5^ spirochetes of either *Bb∆1-18*, *Bb∆19-22* or *Bb∆27-30*. Two additional groups were infected with wild type or *Bb*Δ*lp28-1* clones to serve as controls. For each group, mice were infected for a duration of either 21 or 91 days; these time points were chosen to assess *ospC* expression levels during early and late stages of infection, respectively. On day 21 and 91 post infection, heart and bladder tissue samples were collected for RNA extraction. Ear biopsy tissue was also collected on days 13, 21, 56 and 91 for RNA extraction as a way to assess the *ospC* transcription profile during infection without sacrificing mice. Expression of *ospC* was normalized against the *flaB* housekeeping gene and compared to mRNA of late log phase *in vitro*-grown *B. burgdorferi* wild type.

**Fig. 3:**
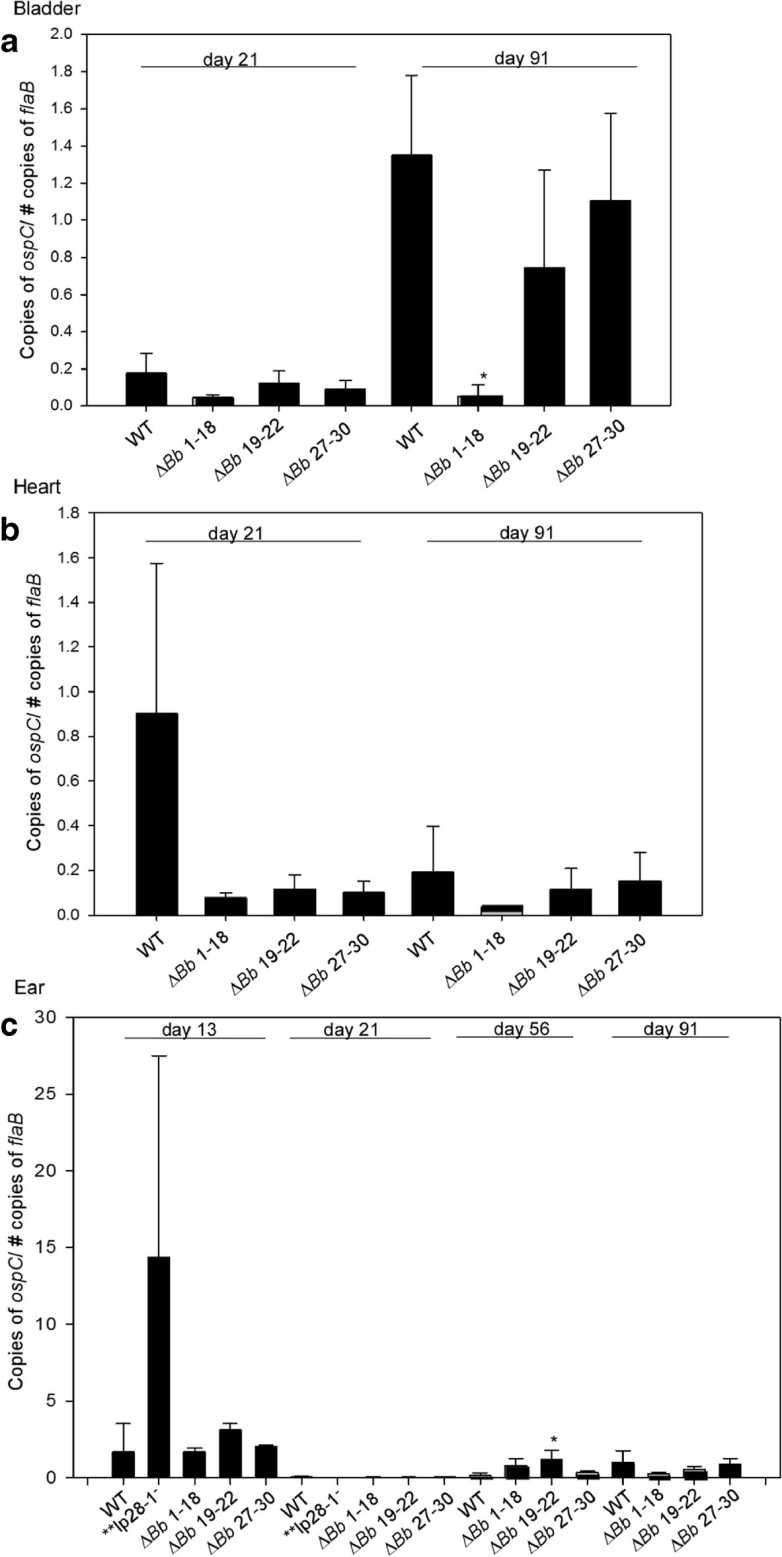
qRT-PCR analysis of *ospC* transcript levels in various tissue sites of infected mice. *ospC* expression profile of spirochetes recovered from the bladder (**a**), heart (**b**), and ear tissue (**c**) of mice infected with wild type, *Bb∆1-18, Bb∆19-22* or *Bb∆27-30* is shown. *ospC* expression was normalized against *flaB* and compared to mRNA of *in vitro*-grown *B. burgdorferi* wild-type cells grown to late log phase. Asterisks show mutant clones with *ospC* expression levels that were statistically different from the wild type using statistical analyses described in Methods. **a**) In bladder tissue, no difference in *ospC* expression was observed at day 21. At day 91, spirochetes from wild type-infected tissues had a statistically significant difference when compared to *Bb∆1-18* infected tissues (*p* = 0.007). **b**) Spirochetes in heart tissue samples showed no significant difference when compared to wild type-infected tissues at both days 21 and 91. **c**) Spirochetes in the ear tissue showed a general decline in *ospC* expression from day 13, which was lowest at day 21 and started to rise at day 56. There was no significant difference in *ospC* expression noted at day 13, 21 and 91. At day 56, spirochetes from mice infected with *Bb*∆*19-22* had a higher expression of *ospC* when compared to those infected by the wild type (p = 0.043). **flaB and *ospC* transcripts were detected at day 13 in tissues collected from mice infected with *Bb*Δ*lp28-1* clone, which correlated with culture results (see Table 3). Data for this clone was not collected after day 21 as all mice successfully cleared infection after this time point

Relative expression levels of *ospC* transcripts were determined by digital droplet PCR (Fig. 3). *FlaB* and *ospC* transcripts were detected at day 13 in tissues collected from mice infected with *Bb*Δ*lp28-1* clone, which correlated with culture results (see Table 3). Data for this clone was not collected after day 21 as all mice successfully cleared infection after this time point. Relative levels of *ospC* expression between tissues infected with the wild type and mutant clones were compared using a Student’s *t*-test. Results presented in Fig. 3a show normalized *ospC* expression from spirochetes in bladder tissue relative to *flaB* transcripts. No significant difference was observed between the wild type and mutant clones recovered from bladder tissues at day 21. At day 91 post infection, *ospC* expression by *Bb*Δ*1-18* spirochetes collected from bladder tissues of mice was significantly repressed relative to that from wild type-infected bladders (*p* = 0.007). No statistical difference was noted with the other clones. Spirochetes recovered from heart tissue at both day 21 and 91 showed no significant difference in *ospC* expression (Fig. 3b).

For ear tissue, normalized *ospC* expression could be determined from day 13 onwards (Fig. 3c). No significant difference in *ospC* expression was observed between wild type and the mutant clones recovered from infected ear tissues collected at day 13, 21 and 91. At day 56 post infection, the *Bb*Δ*19-22* clone was found to have a significantly higher level of *ospC* expression compared to the wild type (*p* = 0.043), while no difference was noted with the other two mutant clones. Taken together, the results from the mice infection studies and RT-qPCR analysis suggest that the lp28-1 regions targeted for deletion are not important for persistent infection by *B. burgdorferi*, and have little to no lasting effects on the expression/repression of *ospC*.

## Discussion

Gene conversion at the *vls* locus on lp28-1 results in antigenic variation of VlsE, which has been demonstrated repeatedly to be essential for immune evasion and persistence by the Lyme disease pathogen [2, 3, 8, 9, 25]. The question remained whether lack of the *vls* locus alone explains the intermediate infectivity phenotype exhibited by clones lacking the lp28-1 plasmid. Previous studies have excluded all remaining genes as having an effect on persistent infection, with the exception of the genetic loci *bbf19-22* and *bbf27-30* [8, 9]. A *B. burgdorferi* mutant lacking *bbf1-18* that was previously reported [8] was included in the present study because previous infection studies were only carried out for 28 days, and the intention of this study was to prolong infection for 91 days to assess long-term persistence. The *bbf1-18* region has several conserved coding regions, short genes, and pseudogenes. In addition, a number of genes in this region (*bbf03, bbf05, bbf10,* and *bbf18*) have been identified as potentially important for infectivity [12]. The *bbf19-22* region encodes one pseudogene (*bbf19*), a conserved hypothetical protein (*bbf20*), a short gene (*bbf21*) and a putative p23 protein (*bbf23)*. Region *bbf27-30* encodes mostly short genes (*bbf27-29*) and one pseudogene (*bbf30*; [20]. Although the possibility has been raised that one or more of these genes could be involved in infectivity or persistence, the data reported here demonstrate that lp28-1 mutant clones lacking *bbf1-18, bbf19-22* or *bbf27-30* were fully capable of tissue dissemination and long-term infection of an immunocompetent murine host. This strongly suggests that these non-*vls* genes of lp28-1 do not play a role in persistent infection by the Lyme disease pathogen.

A previous study suggested that failure of immune evasion by clones lacking lp28-1 might also be due to altered expression of the surface lipoprotein, OspC. It was reported that *ospC* expression by lp28-1^−^ spirochetes was abnormally high, suggesting an impairment in downregulation of this protein [14]. OspC is required for the establishment of mammalian infection and is an effective immune target that has to be downregulated to avoid immune clearance [18, 26, 27]. Regulation of antigen expression has been shown to be essential for spirochete survival within the complex enzootic cycle involving the tick and mammal [16, 23, 28–30]. OspC production increases when the tick feeds [30, 31], and has been shown to be required for *B. burgdorferi* to establish infection in the mammal [26, 27, 32–34]. However, OspC is dispensable during persistent infection [27, 35], and it has been reported that its continued expression in an immunocompetent animal is detrimental to *B. burgdorferi* survival [18]. This suggests that *B. burgdorferi* must downregulate OspC following a specific humoral response as a strategy to evade [28] host immunity and ensure persistence in the mammalian host. Failure of OspC downregulation by spirochetes lacking lp28-1 has been previously shown, and it has been suggested that OspC regulation by genes on this plasmid may be a potential mechanism of immune evasion [14].

Despite the observed ability of the mutant clones in the present study to persistently infect mice, an additional component of this study was to assess whether genes within the *bbf01-22* or *bbf27-30* regions are potentially responsible for the increased *ospC* expression previously observed for spirochetes lacking lp28-1. *FlaB* and *ospC* mRNA transcripts were consistently detected from ear tissues at day 13 post infection. At day 12, it has been shown that *ospC* is abundantly expressed in the skin compared to the heart and joint [17], making the skin a reliable tissue to observe *ospC* expression during early infection without sacrificing the animal. Anti-OspC antibody is detectable at day 10, and then peaks at days 24–45 before starting to decline [15]. Consistent with this timeline, the results showed relatively high levels of *ospC* expression in the ear at day 13 post infection when humoral immunity against OspC is not fully established (Fig. 3c). By day 21 post infection, *ospC* transcript levels from all tissue samples collected from mice infected with mutant or wild type spirochetes were greatly reduced suggesting that the mutations generated within lp28-1 did not affect OspC down regulation. This is indicative of either immune selection against spirochetes that abundantly express this lipoprotein and/or persistence of spirochetes that decrease its expression. Moreover, a general increase in *ospC* expression by both the wild type and mutant clones was observed in infected ear and bladder tissues at days 56 and 91, which coincides with the reported decline of anti-OspC antibody [15] (Fig. 3a and c).

Overall, relatively comparable *ospC* expression levels were observed for the wild type and mutant clones. The wild type clone generally showed higher levels of *ospC* expression, with the exception of day 56 when *Bb*Δ*19-22* mutant spirochetes colonizing ear tissue exhibited significantly higher expression levels. Initial delay in tissue colonization was also observed with this mutant at days 7 and 14 post infection. In the end, the higher *ospC* expression levels did not correspond to clearance of spirochete infection as demonstrated by the long-term mouse infection studies.

From the data presented in this study, the lp28-1 regions targeted for deletion are not important for persistent infection by *B. burgdorferi*, and have little to no lasting effects on the expression/repression of *ospC*. However, it is conceivable that the regions under study may have a detectable phenotype in mice when given a lower inoculum dose. Moreover, it is possible that these regions could play a role in other aspects of infectivity; both possibilities will require further study.

## Conclusions

The data reported herein indicate that regions *bbf1-18*, *bbf19-22* and *bbf27-30* of lp28-1 are not involved in persistent infection by *B. burgdorferi*, and do not critically alter the overall expression levels of *ospC* during murine infection leading to immune clearance. Moreover, along with the previous data involving mutation of non-*vls* loci on lp28-1, these findings suggest that the *vls* locus may be the sole genetic element on this plasmid responsible for immune evasion and persistence exhibited by the Lyme disease pathogen.
